# Non-alcoholic steatohepatitis-related liver tumorigenesis is suppressed in mice lacking hepatic retinoid storage

**DOI:** 10.18632/oncotarget.19978

**Published:** 2017-08-07

**Authors:** Takayasu Ideta, Yohei Shirakami, Masaya Ohnishi, Akinori Maruta, Koki Obara, Tsuneyuki Miyazaki, Takahiro Kochi, Hiroyasu Sakai, Hiroyuki Tomita, Takuji Tanaka, William S. Blaner, Masahito Shimizu

**Affiliations:** ^1^ Department of Gastroenterology, Gifu University Graduate School of Medicine, Gifu, Japan; ^2^ Department of Informative Clinical Medicine, Gifu University Graduate School of Medicine, Gifu, Japan; ^3^ Department of Tumor Pathology, Gifu University Graduate School of Medicine, Gifu, Japan; ^4^ Division of Pathology, Gifu Municipal Hospital, Gifu, Japan; ^5^ Department of Medicine, Columbia University, New York, NY, USA

**Keywords:** retinoid, hepatocellular carcinoma (HCC), non-alcoholic steatohepatitis (NASH), lecithin:retinol acyltransferase (LRAT), oxidative stress

## Abstract

Non-alcoholic fatty liver disease has become one of the most common causes of chronic liver disease that can develop into a more serious form, non-alcoholic steatohepatitis, leading to liver cirrhosis and hepatocellular carcinoma. Although hepatic retinoid stores are progressively lost during the development of liver disease, how this affects steatohepatitis and its related hepatocarcinogenesis is unknown. In order to investigate these, we used subcutaneous injection of streptozotocin (0.2 mg/body) and high-fat diet to induce steatohepatitis and hepatic tumorigenesis in lecithin:retinol acyltransferase -deficient mice (n = 10), which lack stored retinoid in the liver, and control mice (n = 12). At the termination of the experiment (16 weeks of age), the development of hepatic tumors was significantly suppressed in mutant mice compared to controls. Lower serum levels of alanine aminotransferase and decreased hepatic levels of cyclin D1 were observed in mutant mice. Mutant mice exhibited increased levels of retinoic acid-responsive genes, including p21, and decreased oxidative stress as evaluated by serum and liver markers. Our findings are consistent with the conclusion that mutant mice are less susceptible to steatohepatitis-related liver tumorigenesis due to increased retinoid signaling, which is accompanied by up-regulated p21 expression and attenuated oxidative stress.

## INTRODUCTION

Non-alcoholic fatty liver disease (NAFLD) is characterized by a broad spectrum of disease forms, ranging from simple fatty liver through non-alcoholic steatohepatitis (NASH), involving chronic inflammation of the liver and increased risk of development of liver fibrosis, cirrhosis, and hepatocellular carcinoma (HCC) [1]. The clinical importance of NAFLD is illustrated by its high prevalence (6.3–33%, with a median of 20%) in the general population [2] and is associated with increased liver-related mortality and HCC [3].

A number of studies have indicated that the presence of insulin resistance and increased oxidative stress are associated with the progression of NAFLD/NASH [4, 5]. In addition, other factors are important for the development and progression of NAFLD/NASH, including genetic polymorphisms, adipocytokine imbalance, pro-inflammatory cytokines, and gut-derived endotoxins [6]. This has led to the proposal of a ‘two-hit hypothesis’ to explain the development of liver disease. According to the ‘two-hit hypothesis,’ the first hit is represented by elevated hepatic lipid accumulation, after which act with other factors as ‘second hits,’ including oxidative stress and pro-inflammatory cytokines, leading to NASH (6). However, an alternative hypothesis, termed the “multiple parallel-hit hypothesis,” proposes that these factors concurrently, rather than sequentially, give rise to steatohepatitis and fibrosis of NASH (7). It should be also importnat that these factors, especially oxidative stress and chronic inflammation, contribute to develop NASH-related HCC [5, 7].

Retinoids, vitamin A and its derivatives, regulate multiple biological functions, including embryonic development, immune function, reproduction, and vision [8]. All-*trans*-retinoic acid, which activates the nuclear retinoic acid receptors, regulates the transcription of numerous target genes. This accounts for most of the activities of retinoids within the body [9, 10]. Retinoids and their target genes also have a great effect on oxidative stress and inflammation [11]. A recent study using the transgenic mice showed that disruption of retinoid signaling increased oxidative stress and this is associated with NASH and hepatocarcinogenesis [11]. On the other hands, administration of retinoid suppressed the development of NASH and its related liver tumors [11, 12]. Another report have demonstrated that acyclic retinoid, a synthetic retinoid, significantly inhibited the development of NAFLD/NASH-related liver tumors by attenuating chronic inflammation [13].

Hepatic stellate cells (HSCs) represent the major storage site for retinoids in the entire organism with as much as greater than 80% of hepatic retinoids and about 60% of the body’s total retinoid pool being stored in the form of retinyl esters in characteristic HSC lipid droplets [9]. The loss of lipid droplets from HSC along with their retinyl ester content is one of the first events observed in the development of hepatic disease [9]. Lecithin:retinol acyltransferase (LRAT) is the only enzyme responsible for hepatic retinyl ester synthesis as evidenced in *Lrat* knockout (KO) mice that completely lack lipid droplets in HSC and possess tiny amount of hepatic retinoid [14-16]. An association among LRAT, hepatic diseases, and various malignancies has been documented [17-21]. Interestingly, retinoid signaling is increased in *Lrat* KO mice and this is associated with resistance to chemically induced liver tumorigenesis of these mice [19]. However, a specific role for LRAT and retinoid signaling in the development and progression of NASH and their role in the subsequent stages of liver disease leading to liver cancer remains unclear.

In the present study, we examined the effects of endogenous HSC retinoid stores and activation of retinoid signaling on NASH and liver tumor development in wild-type and *Lrat* KO mice that were administered streptozotocin (STZ) and then fed a high-fat diet (HFD) to induce NASH and HCC.

## RESULTS

### General observations

Among the C57BL/6 wild-type (WT) and *Lrat* KO mice, no significant differences were observed in body weight (24.3 ± 4.9 vs. 26.6 ± 5.4 g), liver weight (2.38 ± 1.66 vs. 1.42 ± 0.49 g), relative weight of the livers (0.11 ± 0.10 vs. 0.10 ± 0.02 g), and white adipose tissue (periorchis and retroperitoneum) weight (0.58 ± 0.66 vs. 0.73 ± 0.64 g) at the end of the experiment.

### Effects of lacking hepatic retinoid on NASH-related liver tumorigenesis

**Figure 1:**
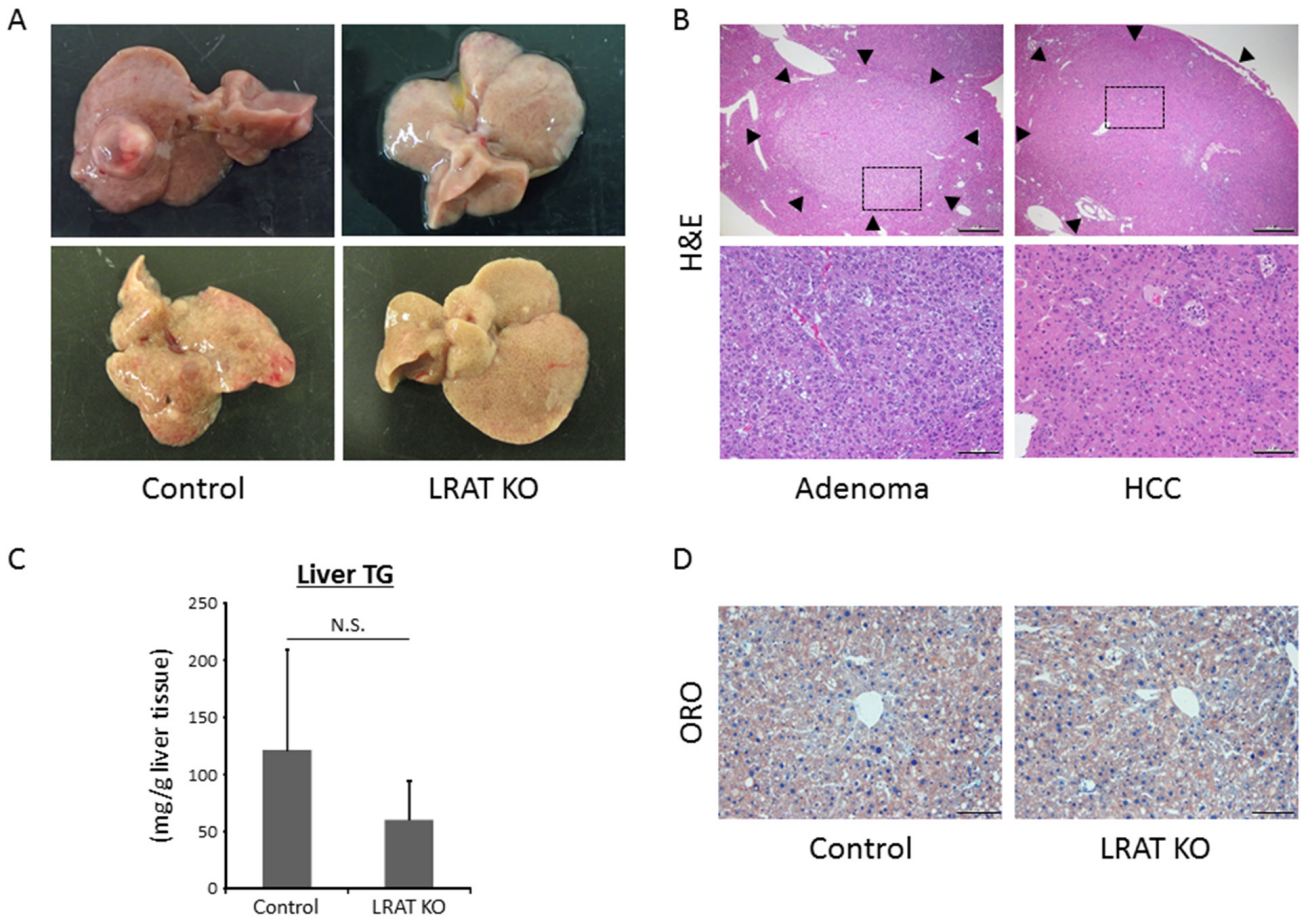
Hepatic neoplastic lesions and steatosis in the experimental mice **(A)** Representative macroscopic images for livers of wild-type control and *Lrat* KO mice. **(B)** Upper; hematoxylin and eosin (H&E) staining of liver tumors (indicated by arrow heads): liver cell adenoma and hepatocellular carcinoma (HCC). Bars, 500 μm. Lower; enlarged pictures of the liver section (enclosed areas with square in Figure 1B upper pictures). Bars, 100 μm. **(C)** Frozen liver sections from wild-type control mice and *Lrat* KO mice were analyzed with Oil red O (ORO) staining to show steatosis. Bar, 100 μm. **(D)** Lipids were extracted from liver samples and hepatic triglyceride (TG) levels were measured in wild-type control (n = 12) and *Lrat* KO mice (n = 10). The values are expressed as the mean ± standard deviation. *P < 0.05 versus control group.

Macroscopically, liver tumors were observed in both mice treated with STZ and HFD (Figure 1A). Microscopically, apparently circumscribed hepatic neoplasms were observed and diagnosed as liver cell adenoma or HCC (Figure 1B). HCC was characterized by trabecular pattern, pseudoglandular, increased nuclear-cytoplasmic ratio, nuclear abnormality, and cellular pleomorphism, while adenoma showed lipid droplets and less cellular pleomorphism. Incidence and multiplicity of liver tumors, including liver cell adenoma and HCC, were significantly lower in *Lrat* KO mice compared to control WT mice (*P* < 0.05, Table 1). With regard to the size of liver tumors, there was no significant difference between WT and *Lrat* KO mice. The average sizes of hepatic adenoma were 1182.9 ± 689.1 and 1056.0 ± 254.6 μm, and HCC were 3975.8 ± 900.1 and 3671.0 ± 1633.4 μm in WT and *Lrat* KO mice, respectively.

**Table 1:** Incidence and multiplicity of hepatic neoplastic lesions in experimental mice

Mice	No. of mice	Incidence (%)		Multiplicity (no. of tumors/mouse)		
		Adenoma	HCC	Adenoma	HCC	Total tumors
						(adenoma and HCC)
Control	12	58	50	1.33 ± 1.56^a^	0.67 ± 0.78	2.00 ± 1.81
LRAT KO	10	10^b^	10^c^	0.35 ± 0.35^c^	0.13 ± 0.35^c^	0.25 ± 0.71^c^

^a^ Mean ± SD.

^b^ Significantly different from control group by chi-square test (P < 0.05).

^c^ Significantly different from control group by Student’s t-test (P < 0.05).

### Effects of lacking hepatic retinoid on the intrahepatic TG levels and biochemical parameters in the experimental mice

In the present study, triglycerides (TG) levels in the liver showed no statistically significant difference among the two groups (Figure 1C). This was consistent with histological findings in the livers of the experimental mice by Oil red O (ORO)-stained liver sections (Figure 1D).

As listed in Table 2, the levels of serum alanine aminotransferase (ALT) were significantly lower for *Lrat* KO mice compared to WT mice, indicating liver inflammation was attenuated in *Lrat* KO mice. On the other hand, other serum parameters, including free fatty acid (FFA), TG, glucose, adiponectin and leptin concentrtions, showed no significant difference between the two groups (Table 2).

**Table 2:** Serum parameters in the experimental mice

	Control	LRAT KO
ALT (IU/l)	111.3 ± 56.1^a^	38.1 ± 13.8^b^
Free fat acid (μEQ/ml)	828.6 ± 149.2	736.3 ± 102.6
Triglyceride (mg/dl)	44.6 ± 23.9	60.5 ± 55.6
Glucose (mg/dl)	386.1 ± 211.8	283.9 ± 187.7
Adiponectin (ng/ml)	2.46 ± 0.01	14.2 ± 17.8
Leptin (ng/ml)	9959 ± 3337	7864 ± 1679

^a^ Mean ± SD.

^b^ Significantly difference from control group by Welch-t test.

### Effects of lacking hepatic retinoid on the expression levels of mRNA and protein involved in inflammation and β-oxidation in the livers of experimental mice

**Figure 2:**
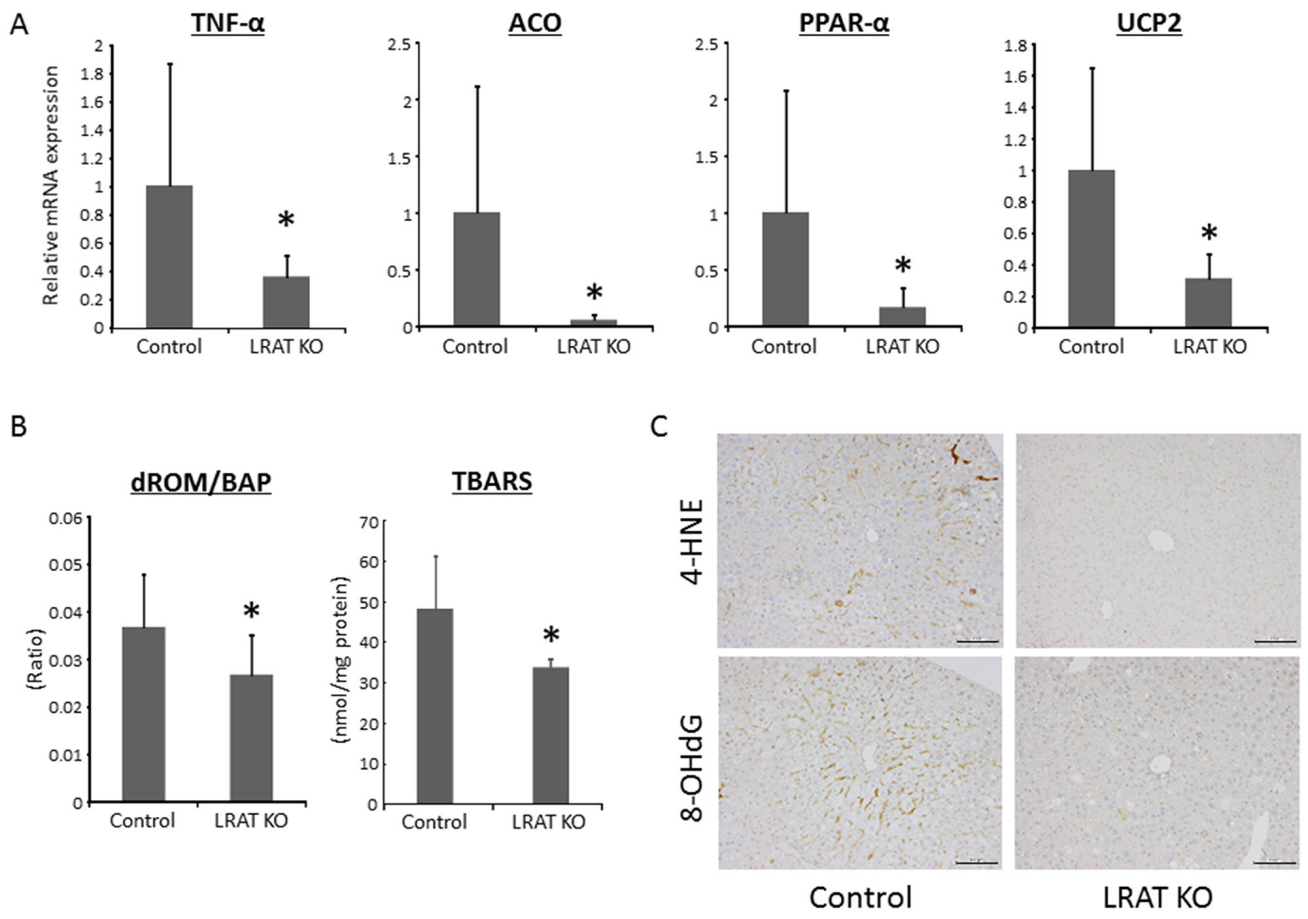
Oxidative stress and expression levels of genes related to inflammation, lipogenesis, and β-oxidation in the liver of experimental mice **(A)** Hepatic expression levels of genes related to inflammation, lipogenesis, and β-oxidation. Total RNA was isolated from the livers of the experimental mice, and the expression levels of acyl-CoA oxidase (Aco), peroxisome proliferator-activated receptor (Ppar)-α, Tnf-α, and uncoupling protein (Ucp) 2 mRNA were examined using quantitative real-time RT-PCR with specific primers. The level of 18S was used as a control. **(B)** The ratio of derivatives of reactive oxygen metabolites (d-ROM) to biological antioxidant potential (BAP) and the levels of thiobarbituric acid reactive substances (TBARS) in the liver tissue were determined in control (n = 12) and *Lrat* KO mice (n = 10). **(C)** Immunohistochemical analyses for 4-hydroxy-2-nonenal (4-HNE) and 8-hydroxy-2’-deoxyguanosine (8-OHdG) in the livers of the experimental mice. Bars, 100 μm. The values are presented as the mean ± standard deviation. *P < 0.05 versus control group.

The mRNA expression level of tumor necrosis factor-α (*Tnf-α*), a gene which is critically involved in inflammation and oxidative stress, was significantly lower in the livers of *Lrat* KO mice compared to WT mice. In addition, the levels of acyl CoA oxidase (*Aco*), peroxisome proliferator-activated receptor-α (*Ppar-α*), and uncoupling protein 2 (*Ucp2*) mRNAs, which are implicated in β-oxidation and oxidative stress, were significantly attenuated in the livers of *Lrat* KO mice (Figure 2A). The protein levels of TNF-α, ACO, PPAR-α, and UCP2 were also investigated, revealing that their levels were reduced in mutants compared to controls (Supplementary Figure 1).

### Effects of lacking hepatic retinoid on oxidative stress in the experimental mice

Serum levels of the derivatives of reactive oxygen metabolites (d-ROMs) and biological antioxidant potential (BAP) were measured to evaluate treatment-induced oxidative stress. The d-ROM/BAP ratio, which indicates the level of oxidative stress, was significantly lower for *Lrat* KO mice compared to WT mice (Figure 2B). The levels of thiobarbituric acid reactive substances (TBARS) in the liver tissue were also markedly reduced in *Lrat* KO mice compared to the control (Figure 2B). In addition, immunohistochemically staining for 4-hydroxy-2-nonenal (4-HNE) and 8-hydroxy-2’-deoxyguanosine (8-OHdG) were lower in the liver of *Lrat* KO mice (Figure 2C), in line with the serum d-ROM/BAP ratio and TBARS levels in the liver. These results suggest that oxidative stress is suppressed in mice lacking hepatic retinoid storage in the present study.

### Effects of lacking hepatic retinoid on hepatic fibrosis and HSC activation in the experimental mice

**Figure 3:**
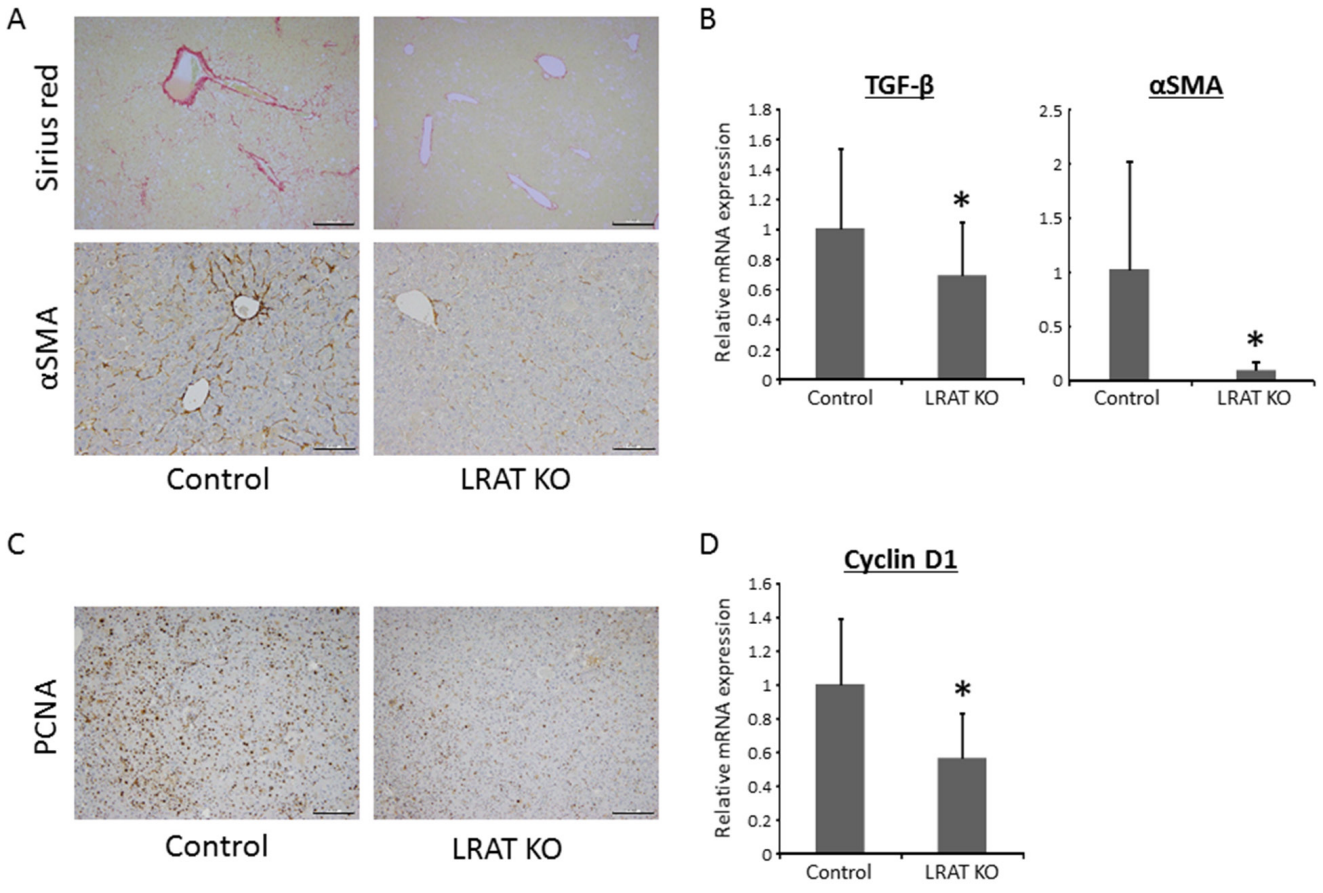
Hepatic fibrosis, HSC activation, and cellular proliferation in the livers of experimental mice **(A)** Sirius Red-staining and immunohistochemical analyses for alpha-smooth muscle actin (α-SMA) and **(C)** proliferating cell nuclear antigen (PCNA) in the livers of the experimental mice. Bars, 200 μm for Sirius Red and PCNA, 100 μm for α-SMA. Total RNA was isolated from the livers of control (n = 12) and *Lrat* KO mice (n = 10), and the expression levels of **(B)** TGF-β, α-SMA, and **(D)** cyclin D1 were examined using quantitative RT-PCR with specific primers. The expression level of 18S rRNA was used as a control. The values are presented as the mean ± standard deviation. *P < 0.05 versus control group.

The Sirius Red-stained liver sections showed fibrosis in WT mice to a greater extent than for the *Lrat* KO mice (Figure 3A). Consistent with the result of this histopathological examination, the mRNA expression levels of transforming growth factor-β (*Tgf-β*), which is known to induce liver fibrosis, were significantly lower than in the livers of *Lrat KO* mice (Figure 3B). To examine activation of HSC, the levels of alpha-smooth muscle actin (α-SMA) were evaluated by immunostaining and mRNA expression. Immunohistochemically staining for α-SMA was lower in the liver of *Lrat* KO mice (Figure 3A), in line with the mRNA expression levels of α-SMA (Figure 3B). These findings indicate that the lack of hepatic retinoid storage in the mutant mice suppresses hepatic fibrosis and HSC activation.

### Effects of lacking hepatic retinoid on cell proliferation in liver tumors of the experiment mice

Positive area for proliferating cell nuclear antigen (PCNA) immunohistochemistry, a useful marker for evaluating cell proliferation, in hepatic tumors was attenuated in *Lrat* KO mice compared to WT mice (Figure 3C). The mRNA expression levels of *Cyclin D1* were also significantly decreased in *Lrat* KO mice liver (Figure 3D), which suggesting that cell proliferation was suppressed in hepatic tumors of the mice lacking hepatic retinoid storage.

### Effects of lacking hepatic retinoid on the protein levels of β-catenin, CYP26A1, p21, and RARβ in the livers of experimental mice

**Figure 4:**
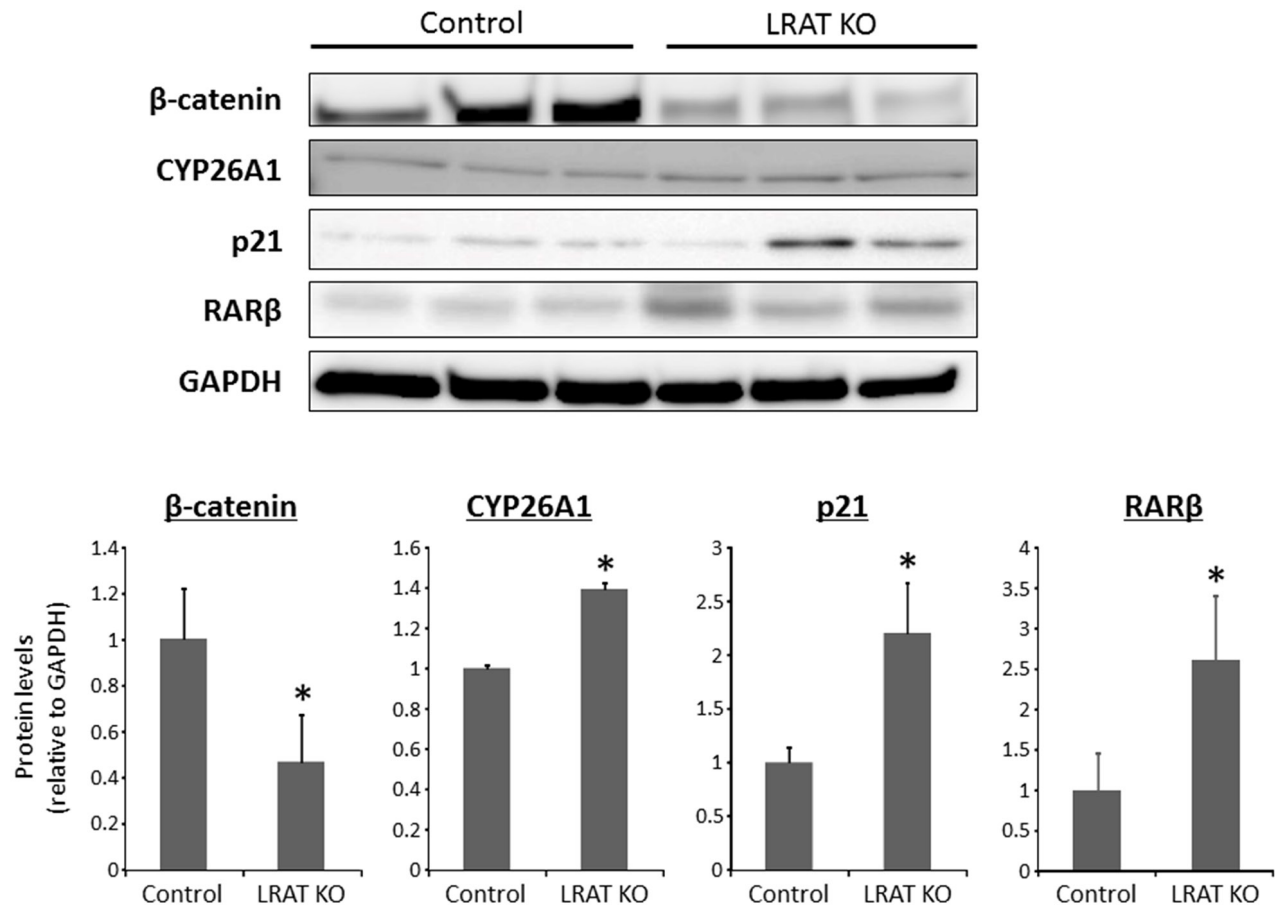
Expression levels of proteins in the livers of the experimental mice Total proteins were extracted from the livers of control and *Lrat* KO mice, and protein levels of β-catenin and CYP26A1, RARβ, and p21 were examined by western blot analysis, using specific antibodies. GAPDH immunostaining served as the loading control. The bar graph shows the mean intensities of each protein. The values are expressed as the mean ± SD. *P < 0.05 versus control group.

The protein levels of β-catenin, CYP26A1, p21, and retinoic acid receptor-beta (RARβ) were determined by western blotting in the livers of *Lrat* KO and WT mice. The levels of β-catenin, a protein that is associated with liver tumorigenesis [22, 23], were significantly lower in the *Lrat* KO group compared to the WT group. On the other hand, protein levels of CYP26A1, p21, and RARβ, known retinoic-acid responsive genes, were higher in the livers of *Lrat* KO mice (Figure 4).

## DISCUSSION

NAFLD/NASH is a serious health issue worldwide since NASH can progress to liver cirrhosis and ultimately cause HCC [24]. The incidence of NASH-related HCC is expected to be on the increase because NAFLD/NASH is closely associated with hypernutrition such as obesity and metabolic syndrome and the number of patients complicating these pathophysiological conditions is growing remarkably [2, 3]. Retinoids are known as key modulators for energy metabolism [10], and the relationship between retinoid abnormalities and liver carcinogenesis has been reported widely [25, 26]. To investigate the role of hepatic retinoid stores in liver carcinogenesis, we employed a genetically modified mouse model that lacks HSC lipid droplets and has only trace amounts of hepatic retinoid, owing to genetically induced *Lrat*-deficiency [16]. In a previous study, we reported that compared to WT mice, *Lrat* KO mice are less susceptible to diethylnitrosamine-induced hepatic tumorigenesis [19]. This was contrary to our original expectation. This earlier study established that *Lrat* KO mice exhibit less hepatic injury, cell proliferation, and cancer initiation at early stages of HCC development [19].

Consistent with this previous report [19], the present study demonstrates that the development of STZ/HFD-induced NASH-associated hepatic neoplasms, both adenoma and HCC, is significantly suppressed in *Lrat* KO mice compared to WT mice. This observation can be explained by several key pieces of data from our present investigation.

First, *Lrat*-deficiency contributes to increased retinoid signaling in the liver, leading to suppressed hepatic tumorigenesis. Up-regulation of retinoic acid-responsive genes, such as CYP26A1 and p21, is indicative of increased retinoid signaling. The reason why *Lrat* KO mice display increased retinoid signaling in the liver appears to be because these mice have higher hepatic levels of retinoic acid, the transcriptionally active retinoid form, due to the decreased conversion of retinol into retinyl esters. This results in more retinol being available for conversion into retinoic acid [16, 19]. Our data suggest that the marked suppression of hepatocarcinogenesis observed in *Lrat* KO mice arises from increased expression of the retinoic acid-responsive p21 gene. It is well established in the literature that the cyclin-dependent kinase inhibitor p21 is regulated by retinoic acid [27, 28] and works to dampen cellular proliferation by promoting cell cycle arrest [29]. RARβ is a predominant retinoid receptor and can also exert the inhibitory effects on cellular proliferation [30]. Among various retinoic acid-responsive genes, RARβ is recognized to act as a tumor suppressor and also have critical roles in the present study for suppressing hepatic carcinogenesis in *Lrat* KO mice.

In this study, *Lrat* KO mice show decreased cellular proliferation as assessed by hepatic PCNA and β-catenin levels and inhibited cell cycle progression as evidenced by a decreased level of cyclin D1 and an increased level of p21. It is known that the Wnt/β-catenin signaling pathway is involved in cell differentiation and proliferation [31] and that deregulation of this pathway is implicated in cellular survival and growth, leading to carcinogenesis including in the liver [23]. The associations between the Wnt/β-catenin pathway and retinoic acid have been described [11, 32, 33]. Loss of retinoid signaling can lead to promotion of the β-catenin and cyclin D1 expressions [11]. Lim *et al.* has reported that retinoic acid is able to suppress cancer stem cell growth via inhibition of Wnt/β-catenin signaling [32]. Recently, it has been demonstrated that retinoic acid suppresses this signaling pathway and ameliorates pancreatic fibrosis [33]. Furthermore, inhibition of Wnt/β-catenin signaling gives rise to suppressing hepatic fibrosis [34, 35]. Although localization and degradation of β-catenin and the other target genes of the signaling were not investigated in detail, increased retinoid signaling appears to inhibit Wnt/β-catenin pathway and to be associated with suppression of hepatic fibrosis and NASH-related liver tumorigenesis in *Lrat* KO mice.

In the present study, hepatic expression levels of PPAR-α, which heterodimerizes with the retinoid X receptor (RXR) to regulate gene transcription, is down-regulated (Figure 2A). This may be thought inconsistent with increased retinoid signaling stated above, but PPAR-α signaling accounts for only a very small percentage of the total retinoid-dependent transcriptional activity in the liver. It is generally accepted that most retinoid-dependent transcription is directly mediated by the retinoic acid receptors (RARs) and all-*trans*-retinoic acid [36]. The changes in retinoid-responsive gene expression we detected arise due to changes in RAR transcriptional regulation not due to PPAR-α/RXR signaling.

A second point that is important for explaining why *Lrat* KO mice display less susceptibility to NASH-related liver cancer is that the absence of *Lrat* is associated with less oxidative stress in the liver, which is a significant contributor to the progression of NAFLD/NASH [7]. Oxidative stress and lipid peroxidation have been implicated in hepatic fibrogenesis and HCC development [1, 37]. Our findings revealed that both of systemic and hepatic oxidative stress in our mice, assessed using the ratio of serum d-ROM to BAP levels and levels of 4-HNE, 8-OHdG, and TBARS in the liver, are significantly lower in *Lrat* KO mice (Figure 2B and 2C). Increased retinoid signaling has been reported to reduce oxidative stress based on studies carried out in a transgenic mouse model where retinoid signaling was inhibited through expression of a dominant-negative form of a retinoid nuclear receptor. These studies convincingly established that in the absence of RA-signaling there is increased oxidative stress and β-oxidation [11].

Oxidative stress is induced, through generation of reactive oxygen species (ROS), by the actions of microsomal CYP2E1 as well as peroxisomal β-oxidation [38, 39]. Down-regulated levels of CYP2E1 in *Lrat* KO liver was observed in our previous study [19]. In addition, decreased hepatic expression of genes related to oxidative stress and peroxisomal β-oxidation, including *Aco*, *Ppar-α,* and *Ucp2,* were observed in *Lrat* KO mice (Figure 2A). In patients with NASH, oxidative phosphorylation is impaired [40, 41] and this impairment is closely associated with the levels of *Ppar-α* and *Ucp2* [42, 43]. Moreover, TNF-α is thought to induce oxidative stress [44] as well, and the hepatic level of *Tnf-a* was decreased in *Lrat* KO mice (Figure 2A). TNF-α is a pro-inflammatory cytokine and an inducer of hepatic fibrosis [45]. *Lrat* KO mice display decreased levels of serum ALT and hepatic *Tnf-a* expression compared to controls, suggesting that chronic inflammation in the liver due to STZ/HFD treatment is ameliorated in *Lrat* KO mice. This too contributes to less liver tumor development. With regard to hepatic fibrosis, Sirius Red-stained liver sections and hepatic expression level of *Tgf-β* (Figure 3A and 3B) indicate that loss of *Lrat* suppresses fibrosis in the liver. HSC activation is also inhibited in mutant mice (Figure 3A and 3B). As discussed above, this difference between *Lrat* KO and WT mice can be attributed to inhibited oxidative stress and decreased TNF-α expression.

One previous study reported that *Lrat* KO mice exhibit no significant difference in their predisposition to liver fibrosis compared to matched WT mice [18]. This discrepancy regarding the sensitivity *Lrat* KO mice to experimentally induced hepatic fibrosis between the present and the previously published study likely arises from differences in responses to the STZ/HFD-treatment, one that induces considerably more oxidative stress and directly affects hepatic retinoid signaling, versus CCl_4_-induced fibrosis which lacks these effects.

In addition to oxidative stress, hepatic steatosis is one of the most important factors to develop NAFLD/NASH [1, 2, 4]. In the present study, however, the fat accumulation and TG levels in liver determined by ORO staining and measuring TG content, respectively, show no significant difference between *Lrat* KO and WT mice (Figure 2C and 2D). This indicates that the lack of hepatic retinoid stores due to *Lrat*-deficiency, at least in this study, has little impact on hepatic steatosis. On the other hand, several factors, including oxidative stress and inflammatory cytokines, are down-regulated in *Lrat* KO mice in comparison to control in the STZ/HFD-treated NASH model (Figure 2). According to the ‘multiple parallel-hit hypothesis,’ several disease-promoting factors act simultaneously and each factor can induce hepatic lipid accumulation (46, 47). If oxidative stress and/or inflammatory cytokines were suppressed, hepatic steatosis would have been also inhibited in *Lrat* KO mice compared to control according to this hypothesis. Therefore, these data are thought to be inconsistent with the ‘multiple parallel-hit hypothesis,’ while the findings above appear to support the ‘two-hit hypothesis’ as lacking *Lrat* has no effect on the ‘first hit’ but exerting suppressive effects on the ‘second hits.’ This we propose contributes to suppressed development of NASH-related liver tumorigenesis we have observed.

In conclusion, *Lrat*-deficient mice, which show a lack of retinoid storage but have an activation of retinoic acid signaling in the liver, exhibited suppressed NASH-related liver inflammation, fibrosis, and tumor development. The underling mechanisms appear to include higher hepatic levels of p21 and decreased oxidative stress due to up-regulated retinoid signaling in the liver.

## MATERIALS AND METHODS

### Animals and chemicals

For all of our studies, we employed male WT and *Lrat* homozygous KO mice congenic in the C57BL/6 genetic background. Genotypes of mice were determined by a polymerase chain reaction (PCR) protocol described previously [14]. STZ was obtained from Sigma Aldrich (Tokyo, Japan). We used High Fat Diet 32 (HFD32) from CLEA Japan (Tokyo, Japan) and CRF-1 from OLIENTAL YEAST (Tokyo, Japan) as the basal diet. HFD32 and CRF-1 contain 560 and 3,245 IU of vitamin A, and 32 and 5.4 g of fat per 100 g diet, respectively. We fully complied with the Guidelines Concerning Experimental Animals issued by the Japanese Association for Laboratory Animal Science and exercised due consideration to minimize pain and suffering. The experimental protocol of this study was also approved by the Institutional Committee of Animal Experiments of Gifu University.

### Experimental procedure and histopathological examination

STZ dissolved in phosphate-buffered saline was administered to the neonatal WT (n = 12) and *Lrat* KO mice (n = 10) within 48 hours after birth as a single subcutaneous injection (0.2 mg/body). After 4 weeks of age, mice were fed HFD32 *ad libitum*. The mouse model was employed with applying to the reported protocol [46]. At the termination of the experiment (at 16 weeks of age), all animals were sacrificed by CO_2_ asphyxiation to analyze serum and hepatic histopathology and biochemical parameters.

The livers were removed immediately after euthanization and the largest hepatic lobe was used for histopathological examination. For all experimental mice, 4-μm-thick sections of formalin-fixed and paraffin-embedded livers were stained with hematoxylin and eosin (H&E) for conventional histopathology and with Sirius red to detect liver fibrosis. Frozen tissue sections were also prepared for ORO staining. Immunohistochemistry was performed using antibodies specific for α-SMA (Abcam, Cambridge, UK), 4-HNE (NIKKEN SEIL, Shizuoka, Japan), 8-OHdG (NIKKEN SEIL) and PCNA (Santa Cruz Biotechnology, Inc., Santa Cruz, CA, USA) [47, 48].

### Clinical chemistry

For chemical analyses, blood samples were collected from the inferior vena cava at sacrifice after 6 hours of fasting. The serum concentrations of glucose (BioVision Research Products, Mountain View, CA, USA), TG (Wako Pure Chemical, Osaka, Japan), FFA (Wako Pure Chemical), adiponectin (BioVendor, Brno, Czech Republic), and leptin (BioVendor) were measured as previously reported [49]. Serum ALT was measured using a standard clinical automatic analyzer (type 7180; Hitachi, Tokyo, Japan). Serum markers for oxidative stress were determined using d-ROMs and BAP tests (FREE Carpe Diem, Diacron International s.r.l., Grosseto, Italy) [47].

### TBARS in the liver tissue of experimental mice

TBARS in the liver samples were measured with an OXI-TEK TBARS assay kit (Enzo Life Sciences, Farmingdale, NY, USA) according to the manufacturer’s instructions.

### RNA extraction and quantitative real-time reverse transcription-PCR analysis

Total RNA was isolated from the livers, which involved microscopic size of tumorous lesions and their surrounding parts, of control (n = 12) and *Lrat* KO (n = 10) mice using the RNeasy Mini Kit (QIAGEN, Venlo, Netherlands). cDNA was synthesized from 0.2 μg of total RNA using the High Capacity cDNA Reverse Transcription Kit (Applied Biosystems, Foster City, CA, USA). Quantitative real-time reverse transcription-PCR (RT-PCR) analysis was performed using a LightCycler Nano (Roche Diagnostics, Indianapolis, IN, USA) with FastStart Essential DNA Green Master (Roche Diagnostics). The sequences of specific primers (Supplementary Table 1) for amplifying *Aco*, *α-sma, Cyclin D1*, *Ppar-α, Tnf-α*, *Ucp2, Tgf-β*, and *18S* genes were previously reported [50] or were obtained using Primer-BLAST (http://www.ncbi.nlm.nih.gov/tools/primer-blast/). The expression level of each gene was normalized to that of *18S*.

### Hepatic lipid analysis

Approximately 200 mg of frozen liver tissue was homogenized, and lipids were extracted using Folch’s method [51]. The TG levels in the liver were measured using the TG E-test Kit (Wako Pure Chemical) as previously reported [52]. To visualize the intrahepatic lipids, ORO staining was utilized based on the standard procedure for frozen liver sections [50].

### Protein extraction and western blot analysis

Total protein was extracted from the livers, which involved microscopic size of tumorous lesions and their surrounding parts, of control and *Lrat* KO mice and equivalent amounts of proteins (10 μg per lane) were examined by western blot analysis [53]. Primary antibodies for β-catenin, CYP26A1, p21, TNF-α, UCP2, and GAPDH were obtained from Cell Signaling Technology (Beverly, MA, USA), and for ACO, PPAR-α, and RARβ were obtained from Abcam (Cambridge, UK). GAPDH served as the loading control. The intensities of the bands were quantified with NIH Image software ver. 1.62.

### Statistical analysis

The results are presented as the means ± standard deviation (SD), and were analyzed using JMP software version 10 (SAS Institute, Cary, NC, USA). Differences among the two groups were analyzed by Welch’s *t-*test for comparison of numbers or chi-square test for comparison of observed frequency. The differences were considered significant at *P* values of less than 0.05.

## SUPPLEMENTARY MATERIALS FIGURE AND TABLE



## Abbreviations

ACO: acyl CoA oxidase
ALT: alanine aminotransferase
BAP: biological antioxidant potential
d-ROM: derivatives of reactive oxygen metabolite
FFA: free fatty acid
HCC: hepatocellular carcinoma
HFD: high-fat diet
4-HNE: 4-hydroxy-2-nonenal
HSC: hepatic stellate cell
LRAT: lecithin:retinol acyltransferase
NAFLD: non-alcoholic fatty liver disease
NASH: non-alcoholic steatohepatitis
8-OHdG: 8-hydroxy-2’-deoxyguanosine
ORO: Oil red O
PCNA: proliferating cell nuclear antigen
PPAR: peroxisome proliferator-activated receptor
RAR: retinoic acid receptor
RXR: retinoid X receptor
SMA: smooth muscle actin
STZ: streptozotocin
TBARS: thiobarbituric acid reactive substance
TG: triglycerides
TGF: transforming growth factor
TNF: tumor necrosis factor
UCP: uncoupling protein
