# Supplementary material for: Non-alcoholic steatohepatitis-related liver tumorigenesis is suppressed in mice lacking hepatic retinoid storage
Source: Oncotarget. 2017 Aug 7;8(41):70695–706. doi: 10.18632/oncotarget.19978 (PMC5642587; doi:10.18632/oncotarget.19978)
Supplement: Supplementary file 1 [file oncotarget-08-70695-s001.pdf]

Non-alcoholic steatohepatitis-related liver tumorigenesis is suppressed in mice lacking hepatic retinoid storage

SUPPLEMENTARY MATERIALS

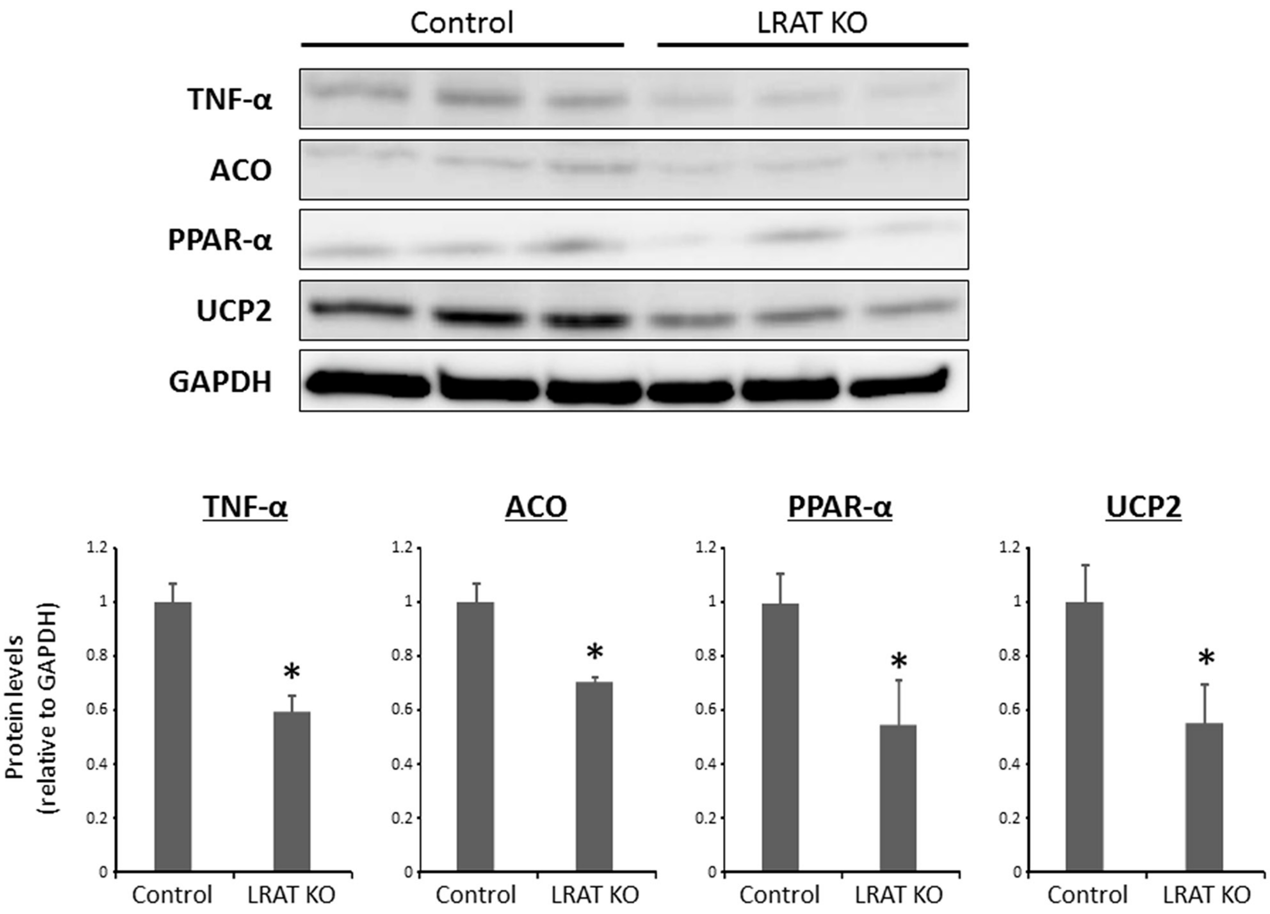

Supplementary Figure 1: The protein levels of genes related to inflammation, lipogenesis, and β-oxidation in the liver of experimental mice. Total proteins were extracted from the livers, and protein levels of TNF-α, acyl-CoA oxidase (ACO), peroxisome proliferator-activated receptor (PPAR)-α, and uncoupling protein (UCP) 2 were examined by western blot analysis, using specific antibodies. GAPDH immunostaining served as the loading control. The bar graph shows the mean intensities of each protein. The values are expressed as the mean ± SD. $^{*}P < 0.05$ versus control group.

Supplementary Table 1: Primers used for qRT-PCR analyses

Genes	Forward primers	Reverse primers
Aco	CACCATTGCCATTCGATACA	TGCGTCTGAAAATCCAAAATC
α-sma	CTCTCTTCCAGCCATCTTTCAT	TATAGGTGGTTTCGTGGATGC
Cyclin d1	TCCCAGACGTTCAGAACC	AGGGCATCTGTAAATACACT
Ppar-α	CTGAGACCCTCGGGGAAC	AAACGTCAGTTCACAGGGAAG
Tgf-β	GGCTACCATGCCAACTT	ACCCACGTAGTAGACGA
Tnf-α	TGGCCCAGACCCTCACACTCAG	ACCCATCGGCTGGCACCACT
Ucp2	ACAGCCTTCTGCACTCCTG	GGCTGGGAGACGAAACACT
18s	CCATCCAATCGGTAGTAGCG	GTAACCCGTTGAACCCCATT
